# Interhemispheric and Intrahemispheric Connectivity From the Left Pars Opercularis Within the Language Network Is Modulated by Transcranial Stimulation in Healthy Subjects

**DOI:** 10.3389/fnhum.2020.00063

**Published:** 2020-03-17

**Authors:** Woo-Kyoung Yoo, Marine Vernet, Jung-Hoon Kim, Anna-Katharine Brem, Shahid Bashir, Fritz Ifert-Miller, Chang-Hwan Im, Mark Eldaief, Alvaro Pascual-Leone

**Affiliations:** ^1^Department of Physical Medicine and Rehabilitation, Hallym University Sacred Heart Hospital, Anyang, South Korea; ^2^Department of Neurology, Harvard Medical School, Boston, MA, United States; ^3^ImpAct Team, Lyon Neuroscience Research Center (CRNL), CNRS UMR5292, INSERM, U1028, University Lyon 1, Bron, France; ^4^Weldon School of Biomedical Engineering, Purdue University, West Lafayette, IN, United States; ^5^Department of Biomedical Engineering, Hanyang University, Seoul, South Korea; ^6^University Hospital of Old Age Psychiatry, University of Bern, Bern, Switzerland; ^7^Department of Neuropsychology, Memory Clinic Zentralschweiz, Lucerne Psychiatry, Lucerne, Switzerland; ^8^Department of Neurophysiology, Neuroscience Center, King Fahad Specialist Hospital, Dammam, Saudi Arabia; ^9^Hinda and Arthur Institute for Aging Research and Center for Memory Health, Hebrew SeniorLife, Boston, MA, United States; ^10^Guttmann Brain Health Institute, Institut Guttmann de Neurorehabilitation, Universitat Autonoma, Barcelona, Spain

**Keywords:** noninvasive brain stimulation, TMS-evoked potentials, gamma band, phase synchronization, continuous theta-burst stimulation

## Abstract

Neural activity related to language can be modulated within widespread networks following learning or in response to disruption—including the experimental application of noninvasive brain stimulation. However, the spatiotemporal characteristics of such modulation remain insufficiently explored. The present study combined transcranial magnetic stimulation (TMS) and electroencephalography (EEG) to explore the modulation of activity across the language network following continuous theta-burst stimulation (cTBS) of the left pars opercularis. In 10 healthy subjects (21 ± 2 years old, four females), neuronavigated cTBS was delivered over the left pars opercularis of the frontal operculum (part of the traditional Broca’s area) at 80% of active motor threshold (AMT) stimulation intensity. Real cTBS and sham cTBS were performed in two different visits separated by at least 48 h. Before, immediately, and 10 min after cTBS, 30 single pulses of TMS were delivered to the left pars opercularis at 80% of the resting motor threshold (RMT), whereas EEG was simultaneously recorded. We examined the cTBS-induced modulation of phase locking values (PLVs) between the TMS-evoked potentials (TEPs) recorded over the pars opercularis and those recorded over its right-hemispheric homolog area, the left supramarginal area, and the left superior temporal area in different EEG frequency bands and different time windows following cTBS. cTBS to the left pars opercularis induced within the gamma band: (1) a significant increase in TEP phase synchronization between the left and right pars opercularis at an early time window (250–350 ms) following cTBS; and (2) significantly increased PLV with the left supramarginal area and the left superior temporal area at a later time window (600–700 ms). In the theta and delta band, cTBS to the left pars opercularis induced significantly increased phase synchronization of TEPs between the left pars opercularis and the posterior left hemispheric language areas at a late time window. In sham condition, there was a significant decrease in TEP phase synchronization of the high beta band between left pars opercularis and left superior temporal area at 200–300 ms. These results contribute to characterize the dynamics of the language network and may have implications in the development of noninvasive stimulation protocols to promote the language rehabilitation in aphasia patients.

## Introduction

Language is processed in widely distributed neural networks including the frontal operculum (Broca’s region) in the dominant hemisphere and its right hemispheric homolog, as well as parietotemporal areas of the left hemisphere (Pulvermüller, 2002; Pulvermüller et al., 2009; Giraud and Poeppel, 2012; Poeppel et al., 2012). Measuring the information flow between those core regions is important for understanding the function of the language network (Singer, 1999; Varela et al., 2001; Buzsaki, 2004). Information flow across nodes of a network can be studied by recording synchronizations of spontaneous cortical oscillations. For instance, a study (Giraud et al., 2007) tried to link auditory structural characteristics of speech and spontaneous brain oscillations within networks important for speech perception and production. Others have examined transient event-related phase synchronizations between two brain areas (Lachaux et al., 1999; Pulvermüller, 2002) or the phase resetting of a cortical oscillation by a perceptual stimulus (Friston et al., 1997; Rizzuto and Kahana, 2001). However, sensory stimuli or cognitive task manipulations can affect in parallel several cortical areas. On the other hand, controlled perturbations applied directly to well-defined brain regions offer a superior strategy to gain insights into the casual interactions between nodes of a given large-scale brain network. Here we applied this strategy to gain insights into the dynamics across the language network.

We applied single pulses of brain magnetic resonance imaging (MRI)-guided (neuronavigated) transcranial magnetic stimulation (TMS) to the pars opercularis of the left frontal operculum and recorded TMS-evoked cortical responses with electroencephalography (EEG) to gain insights into the connectivity dynamics across interhemispheric and intrahemispheric connections of the language network. The use of neuronavigated TMS is important to ensure precise targeting of a given brain region (Gugino et al., 2001; Bashir et al., 2011). We examined the modulation of TMS-evoked cortical responses induced by continuous theta-burst stimulation (cTBS), a noninvasive stimulation protocol that has been shown not only to decrease the excitability of the targeted area (Huang et al., 2005) but also to modulate brain oscillations and synchronizations (Vernet et al., 2013). CTBS can provide longer-lasting modulatory effects with shorter stimulation time than traditional repetitive TMS, which can have important clinical implications.

We hypothesized that such an approach, using task-free and externally triggered oscillations, would provide more direct physiological evidence for connectivity mechanisms. Furthermore, synchronization and desynchronization of these task-free oscillations in response to the plasticity-inducing cTBS protocol could provide information relevant to a future noninvasive intervention for the rehabilitation of patients with aphasia (Miniussi and Thut, 2009). Specifically, we hypothesized that cTBS to the left pars opercularis would change interhemispheric synchronization of TMS-evoked potential (TEP) between the left and the right pars opercularis and intrahemispheric interactions between the left pars opercularis and the left supramarginal and left superior temporal areas.

## Materials and Methods

### Participants

Ten young, healthy, right-handed adults [the average Edinburgh Handedness Inventory (Oldfield, 1971); score, 83] volunteered to participate in the study (21 ± 2 years old; range, 18–24 years old; four females). None of them had a history of psychiatric or neurological conditions, and all had normal neurological and medical examinations, and Mini-Mental State Examination scores in the normal range (Miltner et al., 1999; Burgess and Ali, 2002; Buschman and Miller, 2007; de Diego-Balaguer et al., 2011). Participants visited twice at least 48 h apart for sham and real repetitive TMS (rTMS) session; half of the participants started with the sham session first. Participants were not taking any medication known to affect motor cortical excitability at the time of the study and did not have any contraindications to TMS. All tolerated the TMS without any adverse effect or complication. All gave their written informed consent to the study, which followed international guidelines for the use of TMS (Rossi et al., 2009), had been approved by the local institutional review board (Beth Israel Deaconess Medical Center, Boston, MA, USA), and was conducted in adherence to the Declaration of Helsinki.

### Experimental Setup

All participants underwent a brain MRI using a 3-T GE machine (GE Healthcare, Waukesha WI, USA) with three-dimensional (3D) modified driven-equilibrium Fourier transform protocol–prepared fast spoiled gradient echo sequence (0.94 × 0.94 × 1-mm resolution; echo time = 2.9 ms; flip angle = 15°) to generate a high-resolution anatomical 3D image to guide TMS.

The stimulation setup consisted of a Nexstim stimulator (Nexstim Limited, Helsinki, Finland) for single-pulse TMS and a MagPro stimulator (MagVenture A/S, Farum, Denmark) for the cTBS intervention. The Nexstim eXimia Neuronavigation system was used to ensure that the exact location of the left pars opercularis was targeted as defined by each individual’s brain MRI (Vernet et al., 2013).

Motor-evoked potentials (MEPs) induced by single-pulse TMS were recorded using surface electromyography (EMG) with pre-gelled, disposable Ag/AgCl electrodes attached to the skin. The active electrode was placed over the first dorsal interosseus (FDI) muscle, the reference electrode over the metacarpophalangeal joint, and the ground electrode over the wrist. The EMG signal was acquired at 3 kHz, filtered (10–500 Hz), amplified, displayed, and stored for off-line analysis.

EEG was recorded with a 60-channel TMS-compatible EEG system (eXimia EEG, Nexstim Limited). This system is designed to avoid amplifier saturation after TMS pulse by using a sample-and-hold circuit that keeps the input of the amplifiers constant from 100 μs prestimulus to 2 ms poststimulus (Virtanen et al., 1999).

### Experimental Session

Participants were seated in a comfortable chair, with a headrest, and with their hands supinated and resting on their laps. Participants were monitored for drowsiness and asked to keep their eyes open during TMS (Vernet et al., 2013). Relaxation of the target muscle (FDI muscle) was controlled by continuous EMG monitoring. All participants wore earplugs to protect them from possible acoustic trauma (Rossi et al., 2009) and reduce contamination of TEPs by auditory responses to the clicks produced by the discharge of the TMS coil.

The left M1 optimal scalp location was determined as the scalp location from which single-pulse TMS induced MEPs of maximum peak-to-peak amplitude in the right FDI muscle. Once this optimal spot was identified, the neuronavigation system was used to ensure consistent coil placement and orientation at the optimal spot. Resting motor threshold (RMT) was defined as the lowest stimulus intensity of the Nexstim stimulator capable of inducing MEPs of ≥50-μV peak-to-peak amplitude in at least 5 out of 10 trials. The RMT was obtained to set the stimulation intensity for subsequent single-pulse TMS. Active motor threshold (AMT) was defined as the lowest stimulus intensity of the MagPro stimulator capable of inducing visible twitches in the FDI muscle in half of the trials while the participants maintained a contraction of the FDI muscle at approximately 20% of the maximal voluntary contraction. The AMT was used to set the stimulation intensity for the cTBS protocol.

cTBS was applied to the left pars opercularis with parameters similar to those used by Huang et al. (2005): three pulses at 50 Hz, with an interval of 200 ms between the last pulse of a triplet and the first pulse of a triplet, for a total number of 600 pulses. The intensity was fixed at 80% of AMT. Because of limitations in our experimental setup, the interstimulus interval was 240 ms compared to the interstimulus interval of 200 ms in the original paradigm introduced by Huang et al. (2005). Thus, in our cTBS paradigm, the triplet repetition rate was approximately 4.17 Hz instead of 5 Hz, both frequencies being included in the theta band. Note that previous research from our group has shown that this cTBS protocol can induce clear changes of oscillations within the motor cortex (Vernet et al., 2013).

**Figure 1:**
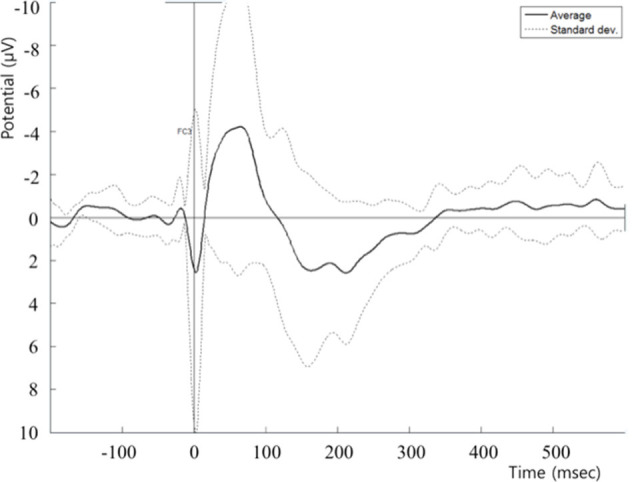
Post cTBS T0 TEP recorded at the FC5 electrode. cTBS, continuous theta-burst stimulation; TEP, transcranial magnetic stimulation (TMS)-evoked potential.

Before cTBS, a series of 30 neuronavigated single pulses of TMS was applied to the left pars opercularis to evoke TEPs (Figure 1). These pulses were applied at an intensity of 80% of RMT. After cTBS, two series of 30 single pulses were applied to the same area and at the same intensity, one immediately after cTBS (T0) and one at 10 min later (T10).

### EEG Data Preprocessing

After recording EEG, the data were preprocessed using an open MATLAB toolbox EEGLAB^1^. Data were band-pass filtered between 1 and 50 Hz. Artifacts induced by transient magnetic fields and eye blinks were eliminated using independent component analysis. The noise-free data were segmented into epochs from 300 ms before the stimulus onset to 1,000 ms after the stimulus onset. Epochs were excluded from further analysis if they contained significant physiological artifacts (amplitude exceeding ±5 μV) in the time range from 50 to 1,000 ms after the stimulus onset at any site over all electrodes.

### Phase Locking Value

Phase locking value (PLV) was used to quantify the functional connectivity between pairs of regions of interest (ROIs). Phase locking value is a well-known index to measure phase synchronization between two time signals acquired from two different electrodes in the same time interval and frequency band (Pulvermüller, 2002). Phase locking value was selected as the functional connectivity measure because it can robustly estimate the interaction between two signals with a constant time delay. Indeed, systematic time delays are expected between the responses to TMS induced at different ROIs. In order to quantify the degree of phase synchrony between two signals *S*_x_(t) and *S*_y_(t), the instantaneous phases *φ*_x_(t) and *φ*_y_(t) were first evaluated using the Hilbert transform. The Hilbert transform of *S(t)* is defined as:

(1) $$\tilde{S}(t)=\frac{1}{\pi }P.V.\int_{\infty }^{\infty }\frac{s(\tau )}{t-\tau }d\tau$$

where $\tilde{S}(t)$ is the Hilbert transform of the time series *S(t)*, and *P.V.* denotes the Cauchy principal value. The instantaneous phase *φ(t)* can then be estimated by:

(2) $$\varphi (t)=arctan\frac{\tilde{S}(t)}{S(t)}$$

The PLV was evaluated using the following definition:

(3) $$\text{PLV}=\left|\langle e^{i\Delta \varphi (t)}\rangle \right|$$

where Δ*φ(t)* = *φ_x_(t)* − *φ_y_(t)*, and |〈⋅〉| is the averaging operator. The PLV ranges from 0 to 1, where the value close to one represents that two signals are synchronized with a constant time lag and the value close to 0 represents that the two signals are temporally independent with each other.

In the present study, PLV features were evaluated for three electrode pairs, FC5-FC6, FC5-CP3, and FC5-CP5, where FC5, FC6, CP3, and CP5 correspond to left pars opercularis, right pars opercularis, left supramarginal area, and left superior temporal area, respectively. The PLV features were evaluated for delta (1–4 Hz), theta (4–8 Hz), alpha (8–13 Hz), low beta (13–21 Hz), high beta (21–30 Hz), and gamma (30–50 Hz) frequency bands using a sliding window with 100-ms width and 50% overlap.

### Statistical Analysis

We employed a Friedman test to evaluate the difference of PLV given different conditions, baseline, T0, and T10, for different frequency bands, different time windows, and different pairs of electrodes. Then, as *post hoc* test, Wilcoxon signed–rank test was utilized to evaluate the difference of PLV measures between conditions. The significance level was set to *p* < 0.05 after multiple-comparisons correction using Bonferroni correction.

## Results

**Figure 2:**
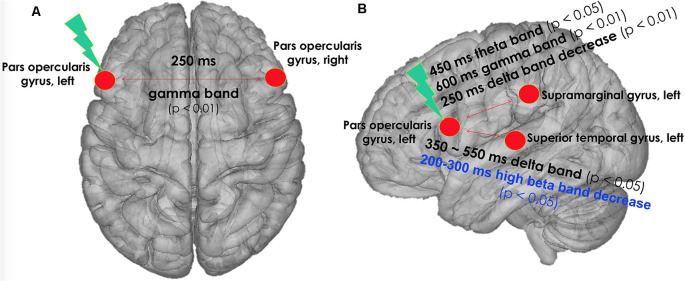
cTBS was applied on left pars opercularis area with three pulses at 50 Hz, with an interval of 200 ms between the last pulse of a triplet and the first pulse of a triplet, for a total number of 600 pulses. The figure indicates changes in phase synchronization of TEP by real cTBS on left pars opercularis area of the language network. **(A)** Top view. **(B)** Left lateral view. Black text: real cTBS condition. Blue text: sham condition. cTBS, continuous theta-burst stimulation; TEP, TMS-evoked potential.

Figure 2 summarizes the significant results of modulation of TEP synchronization induced by cTBS of the left pars opercularis. Please note that no other significant modulations of TEP synchronization across the studied ROIs were found. By EEG frequency bands, the significant findings were as follows:

### Changes in Gamma-Band Synchronization of TEPs in Real cTBS Condition

**Figure 3:**
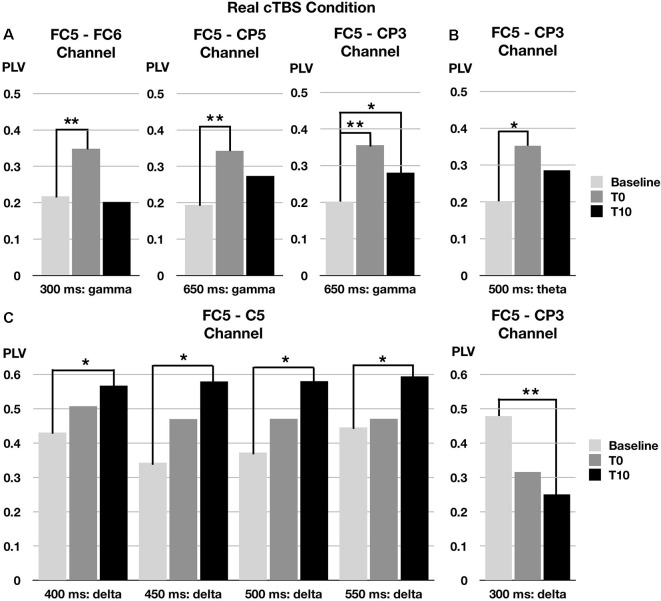
Changes of phase locking value (PLV) by real cTBS on the left pars opercularis. cTBS, continuous theta-burst stimulation. FC5, FC6, CP3, and CP5 correspond to left pars opercularis, right pars opercularis, left supramarginal area, and left superior temporal area, respectively. *p < 0.05, **p < 0.01. **(A)** Gamma band changes. **(B)** Theta band changes. **(C)** Delta band chnages.

In the gamma band (30–50 Hz) at a 250- to 350-ms time window, the phase synchronization of TEPs between the left and right pars opercularis significantly increased at T0 after real cTBS compared to baseline (corrected *p* = 0.009). At a later time window (600–700 ms), the phase synchronization of TEPs in the gamma band between the left pars opercularis and the left supramarginal region significantly increased at both T0 and T10 compared to baseline (corrected *p* = 0.022). Similarly, at the same time window (600–700 ms), the phase synchronization of TEPs in the gamma band between the left pars opercularis and the left superior temporal region significantly increased at T0 (but not T10) compared to baseline (corrected *p* = 0.005; Figure 3A).

### Changes in Theta-Band Synchronization of TEP in Real cTBS Condition

In the theta band (4–8 Hz) in a 450- to 550-ms time window, the phase synchronization of TEPs between the left pars opercularis and the left supramarginal region significantly increased at T0 after real cTBS compared to baseline (corrected *p* = 0.037; Figure 3B).

### Changes in Delta-Band Synchronization of TEP in Real cTBS Condition

In the delta band (1–4 Hz) in a wide time window (350–600 ms), the phase synchronization of TEPs between the left pars opercularis and the left superior temporal area significantly increased at T10 after real cTBS compared to baseline (corrected *p* = 0.009). However, the synchronization of TEPs between the left pars opercularis and left supramarginal in the delta band significantly decreased at an earlier time window (250–350 ms; Figure 3C).

### Changes in Beta2-Band Synchronization of TEP in Sham cTBS Condition

**Figure 4:**
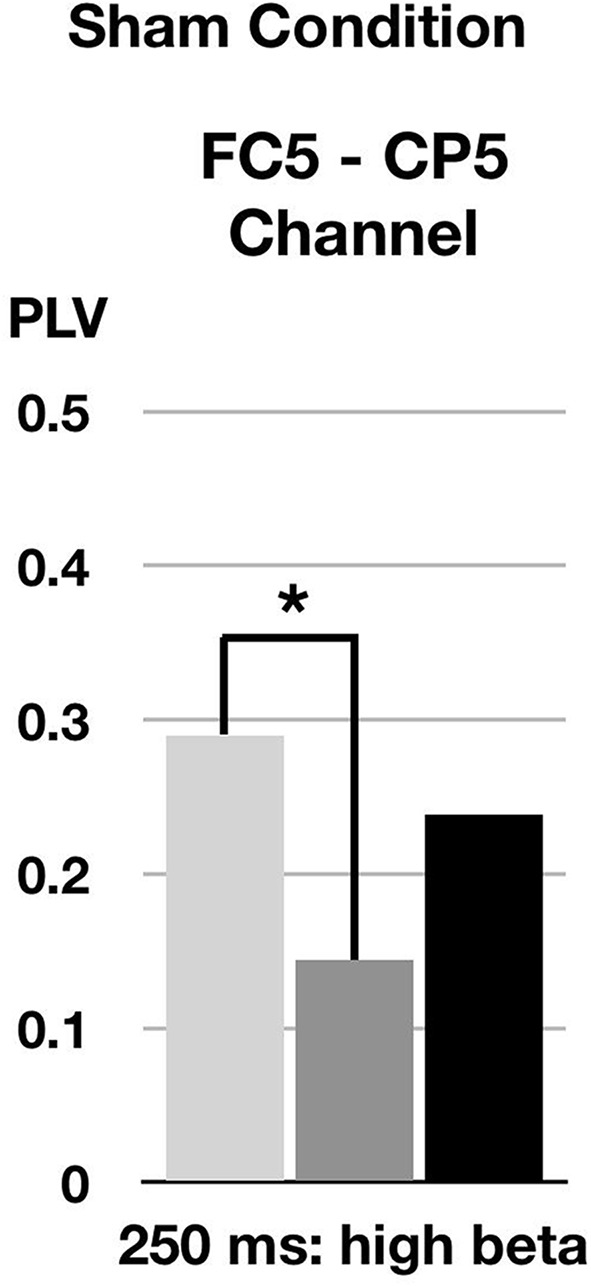
Changes of phase-locking value (PLV) by *sham* cTBS. FC5 and CP5 correspond to left pars opercularis and left superior temporal area, respectively. *p < 0.05.

In the high beta band (21–30 Hz) in a time window (200–300 ms), the phase synchronization of TEPs between the left pars opercularis and the left superior temporal area significantly decreased at T0 after sham cTBS compared to baseline (corrected *p* = 0.014; Figure 4).

## Discussion

cTBS to the left pars opercularis induced significant changes in phase synchronization of TEPs evoked from the left pars opercularis. Specifically, we found: (1) a significant increase in the gamma frequency between the left and right pars opercularis at an early (250–350 ms) time window and a similar increase between the left pars opercularis and the left posterior language areas at a later time period (600–700 ms); and (2) a significant increase in the theta and delta bands between the left pars opercularis and the posterior language areas. These results provide novel insights into the interhemispheric and intrahemispheric dynamics of the language network in healthy young individuals.

cTBS is a TMS protocol that has been primarily used to induce a decrease of excitability in the stimulated area. Indeed, it has been shown that cTBS applied over M1 can induce depression of the MEPs subsequently evoked by single-pulse TMS (Huang et al., 2005). However, recent studies using TMS coupled with EEG, magnetoencephalography (MEG) and/or magnetic resonance spectroscopy have revealed that cTBS also has an impact on local and remote oscillatory systems, on synchronization, and on inhibitory γ-aminobutyric acid (GABA) concentration level (Allen et al., 2014; Du et al., 2018).

Here, we found that cTBS over the left pars opercularis induced an increase of synchronization in the gamma band in early TEPs (250 to 350 ms time window) and in late TEPs (600–700 ms). GABAergic interneurons are known to be the source of the generation of gamma oscillation (Wang and Buzsáki, 1996). Our finding of increased gamma-band synchronization after cTBS might be caused by increased activity of local inhibitory interneurons subtending the inhibitory interaction between the left and right pars opercularis region. Studies using spinal epidural recordings have shown that cTBS applied over M1 suppresses the earliest I1-wave (Di Lazzaro et al., 2005), which is primarily derived from GABA-related interneuronal circuits within the human motor cortex (Ziemann et al., 1998). Thus, cTBS might have decreased cortical excitability in the left pars opercularis and increased inhibition, which led to a change in TMS-evoked EEG gamma synchronization. Alternatively, the increase of gamma coherence might be related to the gamma frequency (50 Hz) embedded in the cTBS pattern.

Interesting deficits of gamma oscillations have been reported in patients with schizophrenia. Ferrarelli et al. (2008) showed that gamma activity evoked by single-pulse TMS applied over the dorsolateral prefrontal cortex was significantly decreased within the first 100 ms after TMS in schizophrenic patients compared to healthy controls. In such patients, spontaneous gamma activity did not show any differences between patients and healthy controls, emphasizing the utility of TMS and specifically the use of TMS–EEG to reveal physiological abnormalities.

Interhemispheric interactions between the left and right pars opercularis regions have been studied as a way to promote language function recovery following stroke. Some have argued that a stroke can impair the normal interhemispheric inhibition balance between the lesioned area and its homolog in the intact hemisphere. If so, decreasing the excitability of the latter area, for example, with noninvasive brain stimulation, might decrease the excessive inhibition it exerts on the former area and thus promote recovery. Consistent with such notions, Naeser et al. (2005) observed significant and persistent improvement in picture-naming abilities in patients with nonfluent aphasia after low frequency rTMS targeting the right frontal operculum. In healthy individuals, similar effects of rTMS on interhemispheric inhibitory interactions between homolog areas of the language network have been shown in studies using positron emission tomography (Thiel et al., 2006), functional MRI (Andoh and Paus, 2011), and behavioral responses (Cappelletti et al., 2008). In the present study, we show for the first time that such interhemispheric interactions might be subtended by synchronization in the gamma band, and that appears possible to modulate it by noninvasive stimulation of the left pars opercularis.

Beyond interhemispheric interactions, our results confirm the relevance of interregional gamma band synchronization within brain networks previously shown in cognition and perception (Sarnthein et al., 1998; Rose and Büchel, 2005; Buschman and Miller, 2007) and language learning (de Diego-Balaguer et al., 2011). For instance, Miltner et al. (1999) showed that intrahemispheric coherence in the gamma band increased during the formation of percepts and memory, linguistic processing, and other behavioral and perceptual functions. Another study showed a greater functional connectivity between the frontal and parietal cortices within the gamma band during successful recollection, as opposed to merely experiencing a feeling of familiarity (Burgess and Ali, 2002). In the language network, an MEG study by Duesburg et al. (2012) reported that a covert verb generation task increased gamma band synchronization among regions activated during the task, in particular, the inferior frontal gyrus and the medial frontal gyrus. A similar result of gamma band phase locking was observed during a silent reading task (Hirata et al., 2010). Our results provide further support for the relevance of phase coherence among gamma rhythms within the language network.

It is also worth noting that local cell assemblies in the cortex are thought to form functional units through synchronous activation in the gamma range (Engel and Singer, 2001; Bastiaansen and Hagoort, 2013). Oscillations may function as nested sets, where lower-frequency activity binds higher-frequency activity (Lisman and Buzsáki, 2008). Theta bursts occur during the up-phase of delta activity (Lakatos et al., 2005), and gamma bursts occur during the up-phase of theta activity (Lisman and Buzsáki, 2008). Thus, it has been suggested that local modules of high-frequency gamma can be functionally linked in temporal synchrony with distant modules *via* lower frequency theta and gamma band regulation. Bastiaansen et al. (2005) showed that the left hemisphere theta activity might be related to the processing of words with extensive semantic stores. The present results, showing increases of gamma TEP synchronization in a late time window (600–700 ms) between the left pars opercularis and the left supramarginal area, may be related to local processes associated with feature binding (Gray and Singer, 1989; Tallon-Baudry et al., 1998), whereas the increases in TEP synchronization in the theta band at the 450 to 550 ms time window between the left pars opercularis area and left supramarginal area might serve as a long-range synchronization between frontal and parietal cortices (von Stein and Sarnthein, 2000; Mellem et al., 2013).

Delta oscillations, on the other hand, have been mainly associated with slow-wave sleep and anesthesia, which are states without conscious awareness. However, delta oscillations might also play a role during wakefulness. For instance, high delta (approximately 3 Hz) appears to organize gamma oscillations during memory retrieval tasks (Burgess and Ali, 2002). Thus, the dissociation of changes in PLV induced by real cTBS within the delta band might be related to different functions and also warrant further study.

We also found a significant decrease in phase synchronization of high beta band in sham condition only between left pars opercularis and left superior temporal areas at T0. Although the PLV was relatively low compared to real cTBS condition, this decrease might have been related to functional changes in this network. There was some evidence that shows decreased coherence in beta band coherence after stimulation of the motor cortex with inhibitory protocol (Mima et al., 2003; Tamura et al., 2005), but also after periods of rest (Fuggetta et al., 2007). Recent evidence also showed a decrease in beta wave at 100–200 ms after sham cTBS (Opitz et al., 2015), in congruence with our sham result. Thus, the decrease in high beta coherence we observed in the sham condition could be related either to a placebo effect of the stimulation, or to the fact that the participants were at rest during the stimulation. We cannot entirely exclude the possible influence of the previous stimulation session due to relatively short washout period (at least 48 h), although studies using a single-session cTBS protocol with usual intensity (such as the one used in the present study) have been reported to last its effects approximately only 20–60 min (Huang et al., 2005; Chung et al., 2016).

### Study Limitations

The present study has a number of limitations that should be considered when interpreting the results. Most importantly, we did not explore the functional correlation of TMS-induced oscillations with language testing. Therefore, it is not possible to experimentally assess whether these oscillation patterns are related to speech production. The left pars opercularis area is well known to be part of the network for speech function. Thus, it seems reasonable to assume that it has a functional role in language, which might be subtended by oscillatory activity similar to the observed TMS-induced oscillations. Nonetheless, this hypothesis awaits future experimental evaluation. In addition, it is important to note that our number of subjects is small, and they were all quite young. Further studies are needed to assess the relevance of the present findings for older adults and elderly individuals. Similarly, we have fewer females than males and an insufficient number of participants to look at gender effects. This too would warrant further studies. Finally, the relevance of our findings to patients with a stroke that might have caused aphasia needs to be examined.

## Conclusion

Our results provide novel insights into the interhemispheric and intrahemispheric dynamics of activity across nodes of the language network and illustrate the potential of using noninvasive brain stimulation for their controlled modulation. Such findings may have implications for the future development of noninvasive stimulation protocols to promote recovery of aphasia.

## Data Availability Statement

The datasets generated for this study are available on request to the corresponding author.

## Ethics Statement

The studies involving human participants were reviewed and approved by Beth Israel Deaconess Medical Center, Boston, MA, USA. The patients/participants provided their written informed consent to participate in this study.

## Author Contributions

W-KY, SB, and AP-L designed the study. A-KB, FI-M, ME, and MV collected the data. W-KY and J-HK analyzed the data. W-KY, MV, J-HK, A-KB, FI-M, ME, SB, and AP-L wrote the main manuscript text. All authors reviewed the manuscript.

## Conflict of Interest

The authors declare that the research was conducted in the absence of any commercial or financial relationships that could be construed as a potential conflict of interest.

## Footnotes

^1^http://sccn.ucsd.edu/eeglab/
