# Corrigendum: Interhemispheric and Intrahemispheric Connectivity From the Left Pars Opercularis Within the Language Network Is Modulated by Transcranial Stimulation in Healthy Subjects

**DOI:** 10.3389/fnhum.2020.00249

**Published:** 2020-07-14

**Authors:** Woo-Kyoung Yoo, Marine Vernet, Jung-Hoon Kim, Anna-Katharine Brem, Shahid Bashir, Fritz Ifert-Miller, Chang-Hwan Im, Mark Eldaief, Alvaro Pascual-Leone

**Affiliations:** ^1^Department of Physical Medicine and Rehabilitation, Hallym University Sacred Heart Hospital, Anyang, South Korea; ^2^Department of Neurology, Harvard Medical School, Boston, MA, United States; ^3^ImpAct Team, Lyon Neuroscience Research Center (CRNL), CNRS UMR5292, INSERM, U1028, University Lyon 1, Bron, France; ^4^Weldon School of Biomedical Engineering, Purdue University, West Lafayette, IN, United States; ^5^Department of Biomedical Engineering, Hanyang University, Seoul, South Korea; ^6^University Hospital of Old Age Psychiatry, University of Bern, Bern, Switzerland; ^7^Department of Neuropsychology, Memory Clinic Zentralschweiz, Lucerne Psychiatry, Lucerne, Switzerland; ^8^Department of Neurophysiology, Neuroscience Center, King Fahad Specialist Hospital, Dammam, Saudi Arabia; ^9^Hinda and Arthur Institute for Aging Research and Center for Memory Health, Hebrew SeniorLife, Boston, MA, United States; ^10^Guttmann Brain Health Institute, Institut Guttmann de Neurorehabilitation, Universitat Autonoma, Barcelona, Spain

**Keywords:** noninvasive brain stimulation, TMS-evoked potentials, gamma band, phase synchronization, continuous theta-burst stimulation

An author's name was incorrectly spelled as “Jeong Hoon Kim.” The correct spelling is “Jung-Hoon Kim.”

The authors apologize for this error and state that this does not change the scientific conclusions of the article in any way. The original article has been updated.

